# Speed of recovery of the most commonly performed shoulder surgeries

**DOI:** 10.1016/j.jseint.2021.03.007

**Published:** 2021-04-21

**Authors:** Florian Grubhofer, Andres R. Muniz Martinez, Lukas Ernstbrunner, Jillian Haberli, Megan E. Selig, Karen Yi, Jon J.P. Warner

**Affiliations:** a Massachusetts General Hospital, Department of Orthopedic Surgery, Harvard Medical School, Boston, MA, USA; b Balgrist University Hospital, Department of Orthopedic Surgery, University of Zürich, Zürich, Switzerland

**Keywords:** Speed of recovery, Shoulder surgery, Reverse total shoulder arthroplasty, Total shoulder arthroplasty, Arthroscopic rotator cuff repair, Arthroscopic biceps tenodesis

## Abstract

**Background:**

Shoulder surgery results in several months of rehabilitation, which is often underestimated by patients preoperatively. Currently, there is little written about this process of recovery. Information on this would help patients to anticipate the trajectory of their recovery. This would also provide a reference point allowing surgeons to compare a patient's progress in their recovery. The purpose of our study was to analyze and document the expected rate of recovery for the most common shoulder operations.

**Methods:**

A retrospective analysis of all patients who underwent total shoulder arthroplasty (TSA), reverse total shoulder arthroplasty (RTSA), arthroscopic rotator cuff repair (ARCR), and arthroscopic biceps tenodesis (BT) using prospectively collected data from the Surgical Outcomes System registry was performed. All patients included had a complete 2-year follow-up data set. The pain score (visual analog scale) was measured preoperatively at 2, 6, and 12 weeks and 6, 12, and 24 months. The American Shoulder and Elbow Surgeons (ASES) and Single Assessment Numeric Evaluation (SANE) score were recorded preoperatively and after 6, 12, and 24 months. The speed of recovery, defined as the percentage of total improvement, for each procedure was assessed as the primary outcome parameter at all time points.

**Results:**

All shoulder interventions resulted in significant improvement of the pain, SANE, and ASES scores 2 years after shoulder surgery. The speed of recovery of all 3 scores was highest after TSA at all measured time points and slowest after ARCR and BT. Measured by the pain score, 90% and 82% of the total improvement after TSA and RTSA was completed after 6 weeks compared to 58% and 59% after ARCR and BT, respectively. Six months postoperatively the ASES recovery rate was significantly higher after arthroplasty (TSA 96% and RTSA 85%) compared to ARCR and BT (76% and 77%, respectively). The SANE score recovery rate was between 82% and 92% (TSA 92%, RTSA 89%, ARCR 87%, BT 82%) 6 months after surgery. After 1 year all patient groups reached 89% or more of the total improvement in all scores, except for the pain after ARCR (89%).

**Conclusion:**

The improvement in pain is fastest after TSA and slowest after ARCR and BT. After TSA and RTSA, >80% of the total pain reduction is achieved 6 weeks postoperatively, whereas after ARCR and BT, >80% of the pain reduction is achieved only 6 months postoperatively. At 12 months postoperatively, the differences in recovery curves were not significant.

Patients often undergo surgery with no clear understanding of the likelihood of a successful outcome and certainly little discussion of the process of their recovery. Moreover, they often compare their expectations to those of friends who may have had an entirely different surgical procedure. Thus, they can neither have realistic expectations nor anticipate important milestones that allow them to plan return to more normal functioning. Porter has presented the concept of value for patients in 3 tiers, with the first being the degree of recovery, the second the process of recovery, and the third the durability of recovery.^17^ We believe that value can be created for patients if they can have a better understanding of their likely trajectory of recovery following a specific shoulder procedure.

While several prior studies have investigated the rehabilitation courses after rotator cuff surgery,^3^^,^^7^^,^^12^^,^^14^^,^^22^ shoulder arthroplasty,^4^^,^^13^^,^^20^ and biceps tenodesis (BT),^1^ none have compared the trajectory of recovery of the most common shoulder surgeries. The purpose of this study was to analyze the process and trajectory of recovery regarding pain, function, and patient satisfaction of several of the most frequently performed shoulder operations. We hypothesized that patients undergoing shoulder arthroplasty will recovery more quickly than those undergoing arthroscopic soft-tissue repair procedures.

## Methods

After obtaining institutional review board approval, we performed a retrospective review of all patients who answered all Surgical Outcomes System Registry (Arthrex, Naples, FL, USA) data questionnaires and had undergone reverse total shoulder arthroplasty (RTSA), total shoulder arthroplasty (TSA), arthroscopic rotator cuff repair (ARCR), and arthroscopic BT between January 2013 and December 2017. This review was performed using prospectively collected patient outcomes in our outcomes registry program. Patients with incomplete data sets or who had complications were excluded. The collected data consisted of the pain score assessed with the visual analog scale,^9^ the American Shoulder and Elbow Surgeons (ASES) score, patient self-assessment,^15^ and the Single Assessment Numeric Evaluation (SANE) score.^23^ The pain score was assessed preoperatively, after 2 and 6 weeks, and after 6, 12, and 24 months. The ASES score and the SANE score were determined after 6, 12, and 24 months. The speed of recovery was determined at each measurement time point as a percentage of the final outcome over the 2-year time course. The assessed 2-year score was considered to be the final outcome.

### Surgical procedure

ARCR and BT were performed under general anesthesia with interscalene block anesthesia in beach chair position. For the rotator cuff repair we used standard techniques appropriate to the tear configuration. All patients who had a rotator cuff repair underwent a concomitant long head BT, except for those who had a previous spontaneous rupture of the long head of the biceps tendon. We performed the arthroscopic BT using a suprapectoral technique. Postoperative management for the rotator cuff repair patients was active and passive immobilization in an abduction pillow for 4-6 weeks, depending on tear size. This was followed with a standard protocol of active range of motion and stretching until 12 weeks after which strengthening was begun. The postoperative course after BT allowed patients to begin immediate passive range of motion exercises but active motion was delayed until 3 weeks post-procedure and strengthening until 8 weeks postoperatively.

Total anatomic shoulder arthroplasty (TSA) and RTSA were performed under general anesthesia with interscalene block anesthesia in beach chair position. All procedures were performed through a standard deltopectoral approach and the arm was immobilized for 4 weeks, though passive range of motion commenced after 1 week. Active motion and then strengthening began at 4 and 8 weeks, respectively.

### Statistics

Normal distribution of data was assessed with the Shapiro-Wilk test. Descriptive data were calculated using mean and standard deviation. Preoperative and postoperative scores were compared with use of the paired t-test (for normal data) or the Wilcoxon signed-rank test (for non-normal data). Differences in scores between time periods and different procedures were compared with one-way analysis of variance (ANOVA; parametric data) and Kruskal-Wallis one-way ANOVA (nonparametric data). Significance was set as *P* < .05 with use of Bonferroni (ANOVA) and Dunn-Bonferroni (Kruskal-Wallis) adjustments and all *P* values were 2-tailed.

## Results

### Patients

Of the 943 performed procedures (217 TSA, 183 RTSA, 149 ARCR, 394 BT), 309 patients met the inclusion criteria. Eighty-four patients (39 females, 45 males, mean age 63 ± 10 years) were treated with TSA, 56 patients (36 females, 20 males, mean age 69 ± 7 years) were treated with RTSA, 40 patients (16 females, 24 males, mean age 58 ± 8 years) were treated with ARCR, 129 patients (62 females, 67 males, mean age 55 ± 11 years) were treated with arthroscopic BT (Table I). Of the 634 excluded patients, 11 patients (1.2%) were excluded due to a complication and the remaining 622 patients (65%) were excluded from the recovery analysis as they had not completed the Surgical Outcomes System data set at all time points.

**Table I:** Demographics of the included patients.

	N (sex)	Age (yr), SD
TSA group	84 (39 f, 45 m)	63 ± 10
RTSA group	56 (36 f, 20 m)	69 ± 7
ARCR group	40 (16 f, 24 m)	58 ± 8
BT group	129 (62 f, 67 m)	55 ± 11

*SD*, standard deviation; *TSA*, total shoulder arthroplasty; *f*, female; *m*, male; *RTSA*, reverse total shoulder arthroplasty; *ARCR*, arthroscopic rotator cuff repair; *BT*, biceps tenodesis (arthroscopic).

### Pain recovery

The mean preoperative pain was 5.3 ± 2.5 (RTSA), 5.9 ± 2.3 (TSA), 4.6 ± 2.4 (BT), and 4.2 ± 2.4 (ARCR). Two years after shoulder surgery the pain was significantly reduced in all groups (*P* < .001).

**Figure 1:**
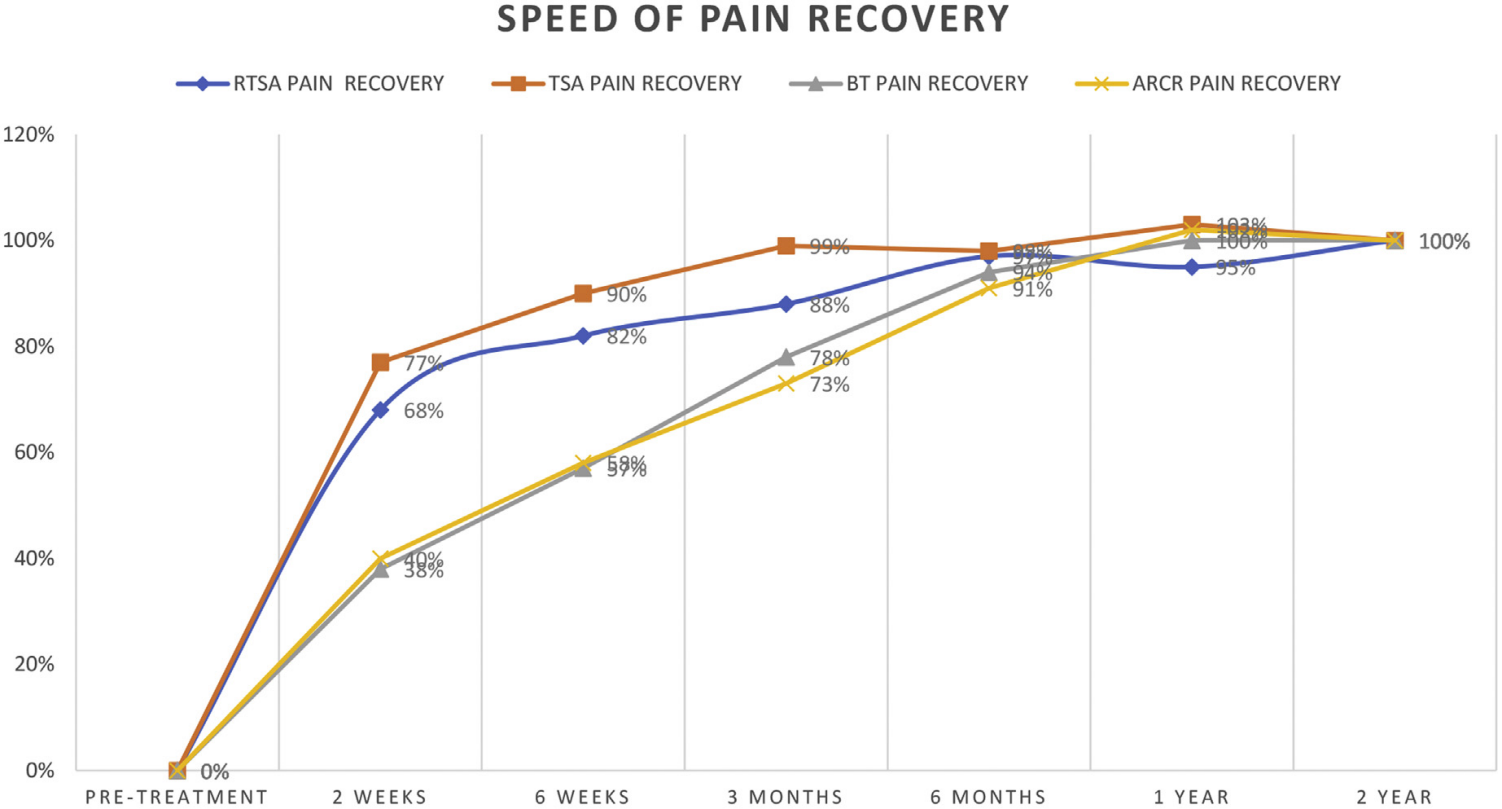
Graphical representation of the speed of pain recovery for a period of 2 years after shoulder surgery. *RTSA*, reverse total shoulder arthroplasty; *TSA*, total shoulder arthroplasty; *BT*, biceps tenodesis; *ARCR*, arthroscopic rotator cuff repair.

The total improvement was highest in the TSA group (4.9 ± 2.6) followed by the RTSA group (4.2 ± 2.9), the BT group (3.3 ± 2.3), and the ARCR group (2.9 ± 2.3). The improvement in pain was at all measured time points highest in the TSA group (Table II and Fig. 1). The recovery rate 2 weeks after TSA was significantly higher compared to the ARCR group (40%, *P* = .033%) and the BT group (38%, *P* < .001). The pain recovery rate 2 weeks after RTSA of 68% was significantly higher compared to the mean recovery rate of the BT group (38%, *P* = .046). The difference between the RTSA group and the ARCR group was not statistically significant (68% vs. 40%, *P* = .604). After 6 weeks the pain recovery was at 90% in the TSA group, which was significantly higher compared to ARCR (58%, *P* = .017) and BT groups (57%, *P* < .001). The improvement rate of the RTSA group (82%) was also significantly higher compared to the ARCR group (58%, *P* = .041) and the BT group (57%, *P* = .031).

**Table II:** Pain recovery values of the 4 different groups.

	RTSA pain	RTSA pain recovery	TSA pain	TSA pain recovery	BT pain	BT pain recovery	ARCR pain	ARCR pain recovery
Pretreatment	5.3 ± 2.5	0%	5.9 ± 2.3	0%	4.6 ± 2.4	0%	4.2 ± 2.4	0%
2 weeks	2.4 ± 1.9	68%	2.1 ± 1.5	77%	3.3 ± 2.2	38%	3.1 ± 2.1	40%
6 weeks	1.8 ± 1.9	82%	1.4 ± 1.8	90%	2.7 ± 2.0	57%	2.6 ± 2.1	58%
3 mo	1.6 ± 1.7	88%	1.0 ± 1.3	99%	2.0 ± 1.9	78%	2.1 ± 1.8	73%
6 mo	1.2 ± 1.6	97%	1.1 ± 1.8	98%	1.4 ± 1.6	94%	1.6 ± 1.9	91%
1 yr	1.3 ± 1.7	95%	0.8 ± 1.1	103%	1.2 ± 1.6	100%	1.3 ± 1.4	102%
2 yr	1.1 ± 1.9	100%	0.9 ± 1.8	100%	1.2 ± 1.7	100%	1.3 ± 1.8	100%
Total improvement	4.2 ± 2.9 (*P*<.001)		4.9 ± 2.6 (*P*<.001)		3.3 ± 2.3 (*P*<.001)		2.9 ± 2.3 (*P*<.001)	

*RTSA*, reverse total shoulder arthroplasty; *TSA*, total shoulder arthroplasty; *BT*, biceps tenodesis (arthroscopic); *ARCR*, arthroscopic rotator cuff repair.

Twelve weeks after TSA the pain improvement rate was 99%, significantly higher compared to the ARCR (73%, *P* < .001) and the BT groups (78%, *P* = .009). The improvement rate 12 weeks after RTSA was 88%, which was not statistically different from the other groups. After 6 months all groups showed a pain recovery rate above 90% (TSA 98%, RTSA 97%, BT 94%, and ARCR 91%). At 1 year follow-up the pain recovery was at or above 100% in the TSA (103%), BT (100%), and RTSA (102%) groups and 95% in the RTSA group. The differences of the improvement rates after 6, 12, and 24 months were not statistically different.

### SANE recovery

**Figure 2:**
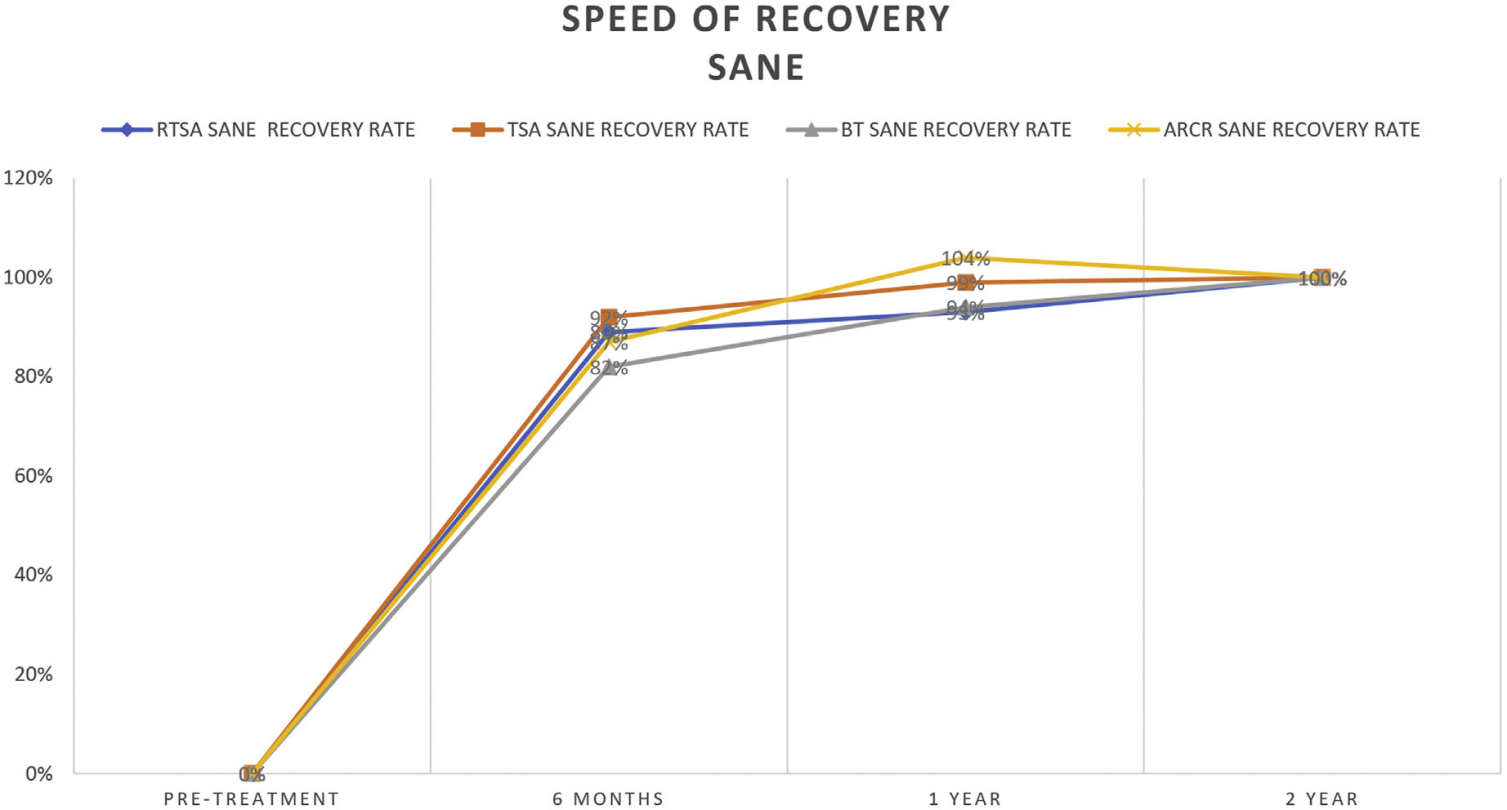
Graphical representation of the speed of SANE recovery for a period of 2 years after shoulder surgery. *SANE*, Single Assessment Numeric Evaluation; *RTSA*, reverse total shoulder arthroplasty; *TSA*, total shoulder arthroplasty; *BT*, biceps tenodesis; *ARCR*, arthroscopic rotator cuff repair.

The preoperative SANE scores showed no significant differences between the 4 intervention groups (RTSA 29, TSA 34, BT 37, ARCR 34). All groups showed a significant improvement in their SANE score 2 years after surgery (all *P* < .001). The highest SANE improvement was observed in the TSA group (51 ± 27) followed by the RTSA group (47 ± 30), the ARCR group (41 ± 24), and the BT group (40 ± 28). The total score improvements were not statistically different between the groups. Six months after TSA the SANE improvement rate was 92%, which was significantly higher compared to the BT group (82%, *P* = .049) followed by the ARCR group (87%, not statistically significant) and the RTSA group (89%, not statistically significant). After 1 year all groups reached an improvement rate of over 93% (RTSA 93%, TSA 99%, BT 94%, and ARCR 104%) (Table III and Fig. 2). The ARCR group showed higher SANE scores at the 1 year follow-up (76 ± 22) compared to the 2 year follow-up (75 ± 2) even though the difference was not significant (*P* = .451).

**Table III:** SANE recovery values of the 4 different groups.

	RTSA SANE	RTSA SANE recovery rate (%)	TSA SANE	TSA SANE recovery rate (%)	BT SANE	BT SANE recovery rate (%)	ARCR SANE	ARCR SANE recovery rate (%)
Pretreatment	29 ± 19	0	34 ± 20		37 ± 20		34 ± 20	
3 mo	NA		NA		54 ± 22	40	47 ± 22	33
6 mo	71 ± 19	89	81 ± 18	92	70 ± 22	82	69 ± 24	87
1 yr	73 ± 24	93	84 ± 19	99	75 ± 25	94	76 ± 22	104
2 yr	76 ± 25	100	85 ± 20	100	77 ± 22	100	75 ± 22	100
Total improvement	47 ± 30		51 ± 27		40 ± 28		41 ± 24	

*SANE*, Single Assessment Numeric Evaluation; *RTSA*, reverse total shoulder arthroplasty; *TSA*, total shoulder arthroplasty; *BT*, biceps tenodesis (arthroscopic); *ARCR*, arthroscopic rotator cuff repair; *NA*, not applicable.

### ASES recovery

**Figure 3:**
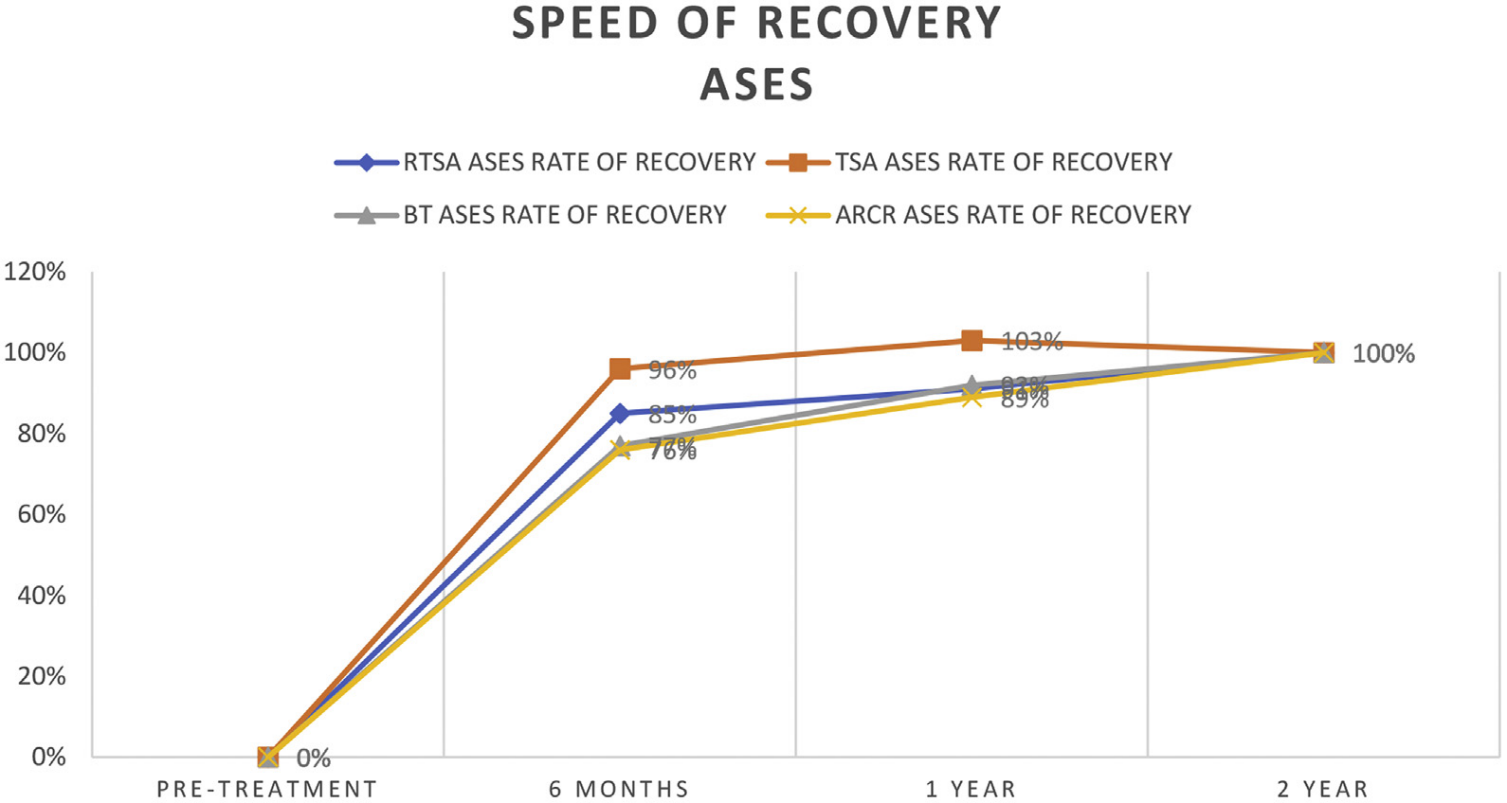
Graphical representation of the speed of ASES recovery for a period of 2 years after shoulder surgery of the 4 different groups. *ASES*, American Shoulder and Elbow Surgeons; *RTSA*, reverse total shoulder arthroplasty; *TSA*, total shoulder arthroplasty; *BT*, biceps tenodesis; *ARCR*, arthroscopic rotator cuff repair.

The total improvement of the ASES score 2 years after shoulder surgery was significant in all 4 treatment groups (*P* < .001). The highest ASES improvement was seen in the TSA group (48 ± 19) followed by the RTSA group (39 ± 22), the BT group (33 ± 20), and the ARCR group (31 ± 18). The total ASES improvement was higher in the TSA group (48 ± 19) than in the RTSA group (39 ± 22) the BT group (33 ± 20) and the ARCR group (31 ± 18). The ASES recovery was fastest in the TSA group, which showed the highest scores at all follow-ups compared to the other groups. Six months after surgery the ASES recovery rate was 96% in the TSA group, which was significantly higher compared to the RTSA group (85%, *P* = .049), the BT group (77%, *P* = .001), and the ARCR group (76%, *P* < .001). At 1 year the TSA group peaked above the total recovery rate, whereas the ASES improvement of the BT group (92%, *P* = .009), the RTSA group (91%, *P* = .001), and the ARCR group (89%, *P* < .001) was still yet to reach the peak (Table IV and Fig. 3).

**Table IV:** ASES recovery.

	RTSA ASES	RTSA ASES rate of recovery	TSA ASES	TSA ASES rate of recovery	BT ASES	BT ASES rate of recovery	ARCR ASES	ARCR ASES rate of recovery
Pretreatment	43 ± 15		40 ± 16		53 ± 17		55 ± 19	
3 mo	NA	NA	NA	NA	66 ± 16	39%	65 ± 17	31%
6 mo	75 ± 14	85%	87 ± 15	96%	78 ± 17	77%	79 ± 18	76%
1 yr	78 ± 17	91%	90 ± 11	103%	83 ± 17	92%	83 ± 16	89%
2 yr	81 ± 17	100%	89 ± 15	100%	89 ± 15	100%	86 ± 16	100%
Total improvement	39 ± 22 (*P*<.001)		48 ± 19 (*P*<.001)		33 ± 20 (*P*<.001)		31 ± 18 (*P*<.001)	

*ASES*, American Shoulder and Elbow Surgeons; *RTSA*, reverse total shoulder arthroplasty; *TSA*, total shoulder arthroplasty; *BT*, biceps tenodesis (arthroscopic); *ARCR*, arthroscopic rotator cuff repair; *NA*, not applicable.

## Discussion

The purpose of this study was to analyze the recovery courses regarding pain, shoulder function, and patient satisfaction of the most common shoulder procedures. It should be explicitly mentioned here that the study did not aim to compare the treatment outcomes of the different shoulder surgeries.

In this study, patients after shoulder arthroplasty realized quicker improvements in pain and patient-reported outcomes compared to patients after ARCR or arthroscopic BT. The recovery parameters after TSA were significantly pronounced compared to those of other shoulder procedures at all measured time points over a 2-year period. After 6 weeks the TSA group showed a 90% pain recovery rate while the arthroscopic tendon repair groups showed pain recovery levels that were far below (BT 57% and ARCR 58%). These differences were substantially attenuated at the 12-week postoperative control appointment and were no longer present at the 6-month control. The total pain improvement 2 years after TSA or RTSA (4.9 ± 2.6 and 4.2 ± 2.9) was higher than after ARCR or BT (2.9 ± 2.3 and 3.3 ± 2.3). This finding should be relativized, however, because preoperative pain scores were higher in the arthroplasty groups than in the ARCR and BT groups. However, as mentioned earlier, the aim of this study has been to highlight the differences in the rehabilitation curves. Comparison of the absolute improvements of the individual shoulder surgeries was intentionally not emphasized in this study, since comparability of the treatment results is not purposeful due to the heterogeneous baseline shoulder conditions. The TSA (63 ± 10 years) group had a statistically significant higher mean age than the ARCR (58 ± 8, *P* = .003) and BT (55 ± 11, *P* < .001) groups. The extent to which age influences the speed of recovery is discussed differently. Regarding postoperative pain recovery after shoulder surgery, a study by Simon et al showed that pain scores during the early rehabilitation phase were higher and pain lasts longer in older patients than in younger patients.^19^ Manaka et al showed that functional recovery after shoulder surgery is also faster in older patients than in younger patients.^14^ However, despite the older age of patients in the TSA group, significantly faster pain recovery and functional recovery were observed in our study than in the significantly younger ARCR and BT patient groups. Possibly the difference in rehabilitation courses would have been even more pronounced if all groups had the same age ranges.

When recovery was measured with the SANE score the highest and fastest improvement was observed in the arthroplasty groups, even though all groups reached the main part of rehabilitation after 6 months. While after 6 months the rehabilitation deficit - measured with the ASES score - was 4% after TSA, a rehabilitation deficit of 24% respectively 23% could be observed in the ARCR and BT group. Faster recovery after shoulder arthroplasty was a surprising finding since it would intuitively seem that arthroplasty is a more invasive procedure than rotator cuff repairs and BT, which were performed arthroscopically in this study.

A major limitation of the study was the high dropout rate of 66%; the reason for this being the strict exclusion of patients who did not complete all data sets at all different postoperative follow-up time points. While a final follow-up in retrospective outcome studies can normally reduce the dropout rate, in this retrospective recovery course analysis it was not possible to obtain missing postoperative data sets. Accordingly, only those patients who completed all data sets at each postoperative follow-up time point were strictly included. Prospective data analysis could help achieve higher response rates in the future. Despite this possible bias, the data collected are of interest and may help to better understand postoperative rehabilitation curves after shoulder surgery. Unfortunately, in this study, the SANE scores and ASES scores for the TSA and RTSA groups were not collected until 6 months, so these scores did not assess the early postoperative phase after TSA and RTSA, which is another limitation of the study. Nevertheless, pain evaluation, which was performed in all groups preoperatively, after 2, 6, and 12 weeks as well as after 6, 12, and 14 months, showed that especially after TSA and RTSA the pain resolved much faster in the early postoperative course compared to the arthroscopic procedures. Another limitation of the study was the use of only 3 patient-related outcome scores to assess the recovery of shoulder procedures. However, this study was intended to examine the rehabilitation process after the most common shoulder surgeries from the patient's point of view. Beyond this, all scores used were validated before.^10^^,^^18^

The recorded recovery curves of the individual scores differed insignificantly per examined procedure. Accordingly, it can be postulated that all 3 collected scores were able to adequately reflect the postoperative course.

Recovery courses after shoulder replacements have been investigated several times. It has also been shown in previous studies that, especially after TSA, the majority of rehabilitation is already completed after 6 months.^8^^,^^11^^,^^20^ The individual recovery curves following ARCR or BT are also comparable to those found in the existing literature.^1^^,^^2^^,^^5^^,^^6^^,^^14^^,^^16^^,^^21^

This study is the first to directly compare postoperative pain, shoulder function, and patient satisfaction courses after the most common shoulder surgeries. While a direct comparison of outcomes after different shoulder surgeries is not useful and was intentionally omitted in this study, direct comparison of rehabilitation courses from the patient's point of view is of high importance. Patients often compare the postoperative course after shoulder surgery with previous shoulder surgeries or with postoperative courses of friends or relatives. This study allows to clarify the differences of the recovery courses depending on the shoulder surgery. The most surprising finding of this direct comparison was that TSA recovery is faster and involves less pain for the patient than after arthroscopic BT or ARCR.

## Conclusion

Direct comparison of recovery curves after TSA, RTSA, ARCR, and BT revealed that TSA demonstrates the fastest rehabilitation in terms of pain, function, and subjective shoulder value. Surprisingly, rehabilitation after BT is more strenuous than rehabilitation after TSA or RTSA due to increased pain and longer rehabilitation time.

## Disclaimers:

*Funding:* No funding was disclosed by the author(s).

*Conflicts of interest:* Jon JP Warner is a Consultant for Wright Medical Group. All the other authors, their immediate family, and any research foundation with which they are affiliated did not receive any financial payments or other benefits from any commercial entity related to the subject of this article.

## References

[1] Agarwalla A., Gowd A.K., Liu J.N., Puzzitiello R.N., Cole B.J., Romeo A.A. (2019). Predictive factors and the duration to pre-injury work status following biceps tenodesis. Arthroscopy.

[2] Altintas B., Anderson N., Dornan G.J., Boykin R.E., Logan C., Millett P.J. (2020). Return to sport after arthroscopic rotator cuff repair: is there a difference between the recreational and the competitive athlete?. Am J Sports Med.

[3] Berglund D.D., Kurowicki J., Giveans M.R., Horn B., Levy J.C. (2018). Comorbidity effect on speed of recovery after arthroscopic rotator cuff repair. JSES Open Access.

[4] Boardman N.D., Cofield R.H., Bengtson K.A., Little R., Jones M.C., Rowland C.M. (2001). Rehabilitation after total shoulder arthroplasty. J Arthroplasty.

[5] Boileau P., Baque F., Valerio L., Ahrens P., Chuinard C., Trojani C. (2007). Isolated arthroscopic biceps tenotomy or tenodesis improves symptoms in patients with massive irreparable rotator cuff tears. J Bone Joint Surg Am Vol.

[6] Chalmers P.N., Erickson B.J., Verma N.N., D'Angelo J., Romeo A.A. (2018). Incidence and return to play after biceps tenodesis in professional baseball players. Arthroscopy.

[7] Collin P., Abdullah A., Kherad O., Gain S., Denard P.J., Ladermann A. (2015). Prospective evaluation of clinical and radiologic factors predicting return to activity within 6 months after arthroscopic rotator cuff repair. J Shoulder Elbow Surg.

[8] Davis D.E., Zmistowski B., Patel M.S., Girden A., Padegimas E., Ramsey M.L. (2020). Predicting postoperative course after total shoulder arthroplasty using a medical-social evaluation model. J Am Acad Orthop Surg.

[9] Delgado D.A., Lambert B.S., Boutris N., McCulloch P.C., Robbins A.B., Moreno M.R. (2018). Validation of digital visual analog scale pain scoring with a traditional paper-based visual analog scale in adults. J Am Acad Orthop Surg Glob Res Rev.

[10] Gilbart M.K., Gerber C. (2007). Comparison of the subjective shoulder value and the Constant score. J Shoulder Elbow Surg.

[11] Kiet T.K., Feeley B.T., Naimark M., Gajiu T., Hall S.L., Chung T.T. (2015). Outcomes after shoulder replacement: comparison between reverse and anatomic total shoulder arthroplasty. J Shoulder Elbow Surg.

[12] Kurowicki J., Berglund D.D., Momoh E., Disla S., Horn B., Giveans M.R. (2017). Speed of recovery after arthroscopic rotator cuff repair. J Shoulder Elbow Surg.

[13] Levy J.C., Everding N.G., Gil C.C., Stephens S., Giveans M.R. (2014). Speed of recovery after shoulder arthroplasty: a comparison of reverse and anatomic total shoulder arthroplasty. J Shoulder Elbow Surg.

[14] Manaka T., Ito Y., Matsumoto I., Takaoka K., Nakamura H. (2011). Functional recovery period after arthroscopic rotator cuff repair: is it predictable before surgery?. Clin Orthop Relat Res.

[15] Michener L.A., McClure P.W., Sennett B.J. (2002). American Shoulder and Elbow Surgeons Standardized Shoulder Assessment Form, patient self-report section: reliability, validity, and responsiveness. J Shoulder Elbow Surg.

[16] Mijic D., Kurowicki J., Berglund D., Rosas S., McNeely E., Motisi M. (2020). Effect of biceps tenodesis on speed of recovery after arthroscopic rotator cuff repair. JSES Int.

[17] Porter M.E. (2010). What is value in health care?. N Engl J Med.

[18] Schmidt S., Ferrer M., Gonzalez M., Gonzalez N., Valderas J.M., Alonso J. (2014). Evaluation of shoulder-specific patient-reported outcome measures: a systematic and standardized comparison of available evidence. J Shoulder Elbow Surg.

[19] Simon C.B., Riley J.L., Coronado R.A., Valencia C., Wright T.W., Moser M.W. (2016). Older age as a prognostic factor of attenuated pain recovery after shoulder arthroscopy. PM R.

[20] Simovitch R.W., Friedman R.J., Cheung E.V., Flurin P.H., Wright T., Zuckerman J.D. (2017). Rate of improvement in clinical outcomes with anatomic and reverse total shoulder arthroplasty. J Bone Joint Surg Am Vol.

[21] van der Meijden O.A., Westgard P., Chandler Z., Gaskill T.R., Kokmeyer D., Millett P.J. (2012). Rehabilitation after arthroscopic rotator cuff repair: current concepts review and evidence-based guidelines. Int J Sports Phys Ther.

[22] Weber A., Paraparan R., Lam P.H., Murrell G.A.C. (2019). Return to sport at 6 months after shoulder surgery. Orthop J Sports Med.

[23] Williams G.N., Gangel T.J., Arciero R.A., Uhorchak J.M., Taylor D.C. (1999). Comparison of the single assessment numeric evaluation method and two shoulder rating scales. Outcomes measures after shoulder surgery. Am J Sports Med.

